# Thank you to the outgoing Editor in Chief of Epilepsia Open, Aristea Galanopoulou

**DOI:** 10.1002/epi4.13003

**Published:** 2024-07-31

**Authors:** International League Against Epilepsy

On September 1, 2024, Aristea Galanopoulou will complete her 8‐year term as the first Editor‐in‐chief of Epilepsia Open. In 2016, the International League against Epilepsy (ILAE) decided to create a new journal to increase the diversity of articles it could publish about epilepsy and to extend the reach of these articles through the open access model. Taking the reins of a completely new journal is a particularly courageous endeavor: there is no track record, no impact factor, and no apparent incentive for authors to submit their work. Dr Galanopoulou embraced this challenge with incredible energy and ingenuity, initially aided by co‐editors Gary Mathern, Dieter Schmidt and Xuefeng Wang and latterly Dong Zhou. At a time when Open Access journals were increasing, she solicited first authors, convincing them of the merits of publishing original articles and reviews in an open access ILAE journal, over a wide range of clinical and basic science topics. Although submissions were slow over the first year or two, as the name became known, numbers of submitted papers began to increase. A major hurdle was obtaining an Impact Factor, a critical step in attracting submissions and establishing a journal. But obtaining an IF is not a simple matter. The editor‐in‐chief has to overcome many obstacles and demonstrate the seriousness and the quality of the journal. A remarkably high first IF of 4.026 was obtained in 2022, consecrating the success of the journal with 400 000 downloaded articles.

Dr. Galanopoulou has worked tirelessly during her 8 years as Editor in Chief at the development and improvement of Epilepsia Open. It is a major tribute to her efforts that the journal is now well‐established, with high recognition among the epilepsy community and with an Impact Factor which among epilepsy journals is second only to Epilepsia's, publishing six issues per year. She has also steered the publication of several special supplements dealing in particular with the development of Common Data Elements for translational epilepsy research, an area that has been at the center of Dr. Galanopoulou's own research and that has high priority for the international epilepsy community. The ILAE is extremely grateful to Dr. Galanopoulou for her profound and fruitful dedication to the establishment of Epilepsia Open.

## Data Availability

Data sharing not applicable to this article as no datasets were generated or analysed during the current study.

